# How round is a protein? Exploring protein structures for globularity using conformal mapping

**DOI:** 10.3389/fmolb.2014.00026

**Published:** 2014-12-09

**Authors:** Joel Hass, Patrice Koehl

**Affiliations:** ^1^Department of Mathematics, University of California, DavisDavis, CA, USA; ^2^Department of Computer Science and Genome Center, University of California, DavisDavis, CA, USA

**Keywords:** proteins, genus zero surfaces, conformal mapping, diffeomorphism, triangular mesh

## Abstract

We present a new algorithm that automatically computes a measure of the geometric difference between the surface of a protein and a round sphere. The algorithm takes as input two triangulated genus zero surfaces representing the protein and the round sphere, respectively, and constructs a discrete conformal map *f* between these surfaces. The conformal map is chosen to minimize a symmetric elastic energy *E*_*S*_(*f*) that measures the distance of *f* from an isometry. We illustrate our approach on a set of basic sample problems and then on a dataset of diverse protein structures. We show first that *E*_*S*_(*f*) is able to quantify the roundness of the Platonic solids and that for these surfaces it replicates well traditional measures of roundness such as the sphericity. We then demonstrate that the symmetric elastic energy *E*_*S*_(*f*) captures both global and local differences between two surfaces, showing that our method identifies the presence of protruding regions in protein structures and quantifies how these regions make the shape of a protein deviate from globularity. Based on these results, we show that *E*_*S*_(*f*) serves as a probe of the limits of the application of conformal mapping to parametrize protein shapes. We identify limitations of the method and discuss its extension to achieving automatic registration of protein structures based on their surface geometry.

## 1. Introduction

Proteins, the end products of the information encoded in the genome of any organism, play a central role in defining the life of this organism. They catalyze most biochemical reactions within cells and are responsible, among other functions, for the transport of nutrients and for signal transmission within and between cells. As a consequence, a major focus of bioinformatics is to study how the information contained in a gene is decoded to yield a functional protein (Pevsner, 2009). The overall principles behind this decoding are well understood. The sequence of nucleotides that forms a gene is first translated into an amino acid sequence, following the rules encoded in the genetic code. The corresponding linear chain of amino acids becomes functional only when it adopts a three-dimensional shape, the so-called tertiary, or native structure of the protein. This is by no means different from the macroscopic world: most proteins serve as tools in the cell and as such either have a defined or adaptive shape to function, much as the shapes of the tools we use are defined according to the functions they need to perform.

Protein structures come in a large range of sizes and shapes. They can be divided into four major groups, corresponding to *fibrous* proteins, *membrane* proteins, *globular* proteins, and *disordered* proteins. Fibrous proteins are elongated molecules in which the secondary structure forms the dominant structure (Fraser, 2012). They are insoluble, play a structural or supportive role in the cell, and are also involved in movement (such as in muscle and ciliary proteins). Membrane proteins are restricted to the phospho-lipid bilayer membrane that surrounds the cell and many of its organelles (White and Wimley, 1999). These proteins cover a large range of shapes, from globular proteins anchored in the membrane by means of a tail, to proteins that are fully embedded in the membrane. Globular proteins, also referred to as *spheroproteins*, due to their compactness, have a unique structure derived from a non-repetitive sequence. They range in size from one to several hundred residues, and adopt a compact structure (Lim, 1974; Levitt and Chothia, 1976; Branden and Tooze, 1991). While proteins belonging to these three groups illustrate the shape-defines-function rule mentioned above, intrinsically disordered proteins form a significant group of exceptions, as they lack stable structures (Dyson and Wright, 1999, 2005; Dunker et al., 2008). Shape remains important for those proteins, although it is its flexibility and plasticity that is of essence, as shown for example in the case of P53 (Oldfield et al., 2009).

The overall importance of shapes for proteins underlines the importance of being able to study, measure and compare those shapes. The most relevant mathematical fields for this topic are Topology and Geometry. One of the first questions that arise in these fields is what distinguishes a space from the simplest and most symmetric shape, the sphere (Bryant and Sangwin, 2011). The 3-dimensional Poincare conjecture for example, recently proved by Perelman (for review see Morgan, 2005), states that if a closed 3-manifold is simply connected then it is homeomorphic to the 3-sphere. In differential geometry, a main focus is how the local geometry of a space, as measured through its curvature, differs from the local geometry of a sphere, and how that difference affects global properties of the space. The Sphere Theorem of differential geometry states that a simply-connected smooth manifold whose curvatures are sufficiently close to those of a sphere is itself a sphere (Brendle and Schoen, 2009).

The fundamental question that arises is how to describe the geometry of a shape such as a protein. The configuration of atoms that constitute a protein can be explicitly obtained by high-resolution experimental methods such as X-ray crystallography, nuclear magnetic resonance (NMR) spectroscopy, or cryo-electron microscopy. As of September 2014, the geometries of over 100,000 proteins are available in the Protein Data Bank (PDB) (Bernstein et al., 1977; Berman et al., 2000). The PDB file corresponding to a protein contains the coordinates of all its atoms. This representation has its limitations. Indeed, it corresponds to a rigid representation of a protein, while proteins have dynamic structures, a key feature that explains their functions, over a large range of time scales, from the nanosecond to the minute time scales (Henzler-Widman and Kern, 2007; Henzler-Widman et al., 2007; Vendruscolo and Dobson, 2011). This means that modeling them with a single rigid representative in 3-space ℝ^3^ can be problematic.

One approach to overcoming the challenges raised by flexibility is to work with the geometry of a 2-dimensional surface that encloses the protein, rather than with the 3-dimensional atomic coordinates. Following the space-filling models such as those of Corey-Pauling-Koltun (CPK; Corey and Pauling, 1953; Koltun, 1965), a protein is represented as the union of balls, whose centers match with the atomic centers and radii defined by van der Waals parameters. The structure of a protein is then fully defined by the coordinates of these centers, and the radii values. One option for generating a 2-dimensional surface that encloses a protein is to consider the geometric surface or boundary of its union of balls, the vdW surface of the protein. Note that other definitions are possible, such as the accessible surface (Lee and Richards, 1971), the molecular surface (Richards, 1977), and the skin surface (Edelsbrunner, 1999). While the dynamics of a protein can cause some distortion of its surface, the geometry of this surface is generally well preserved under motions, much more so than the occupied solid region in 3-space. Focusing attention on the surface of the boundary of a protein is also biologically reasonable, since the main biological functions of a protein take place at its surface.

Within this framework, the basic question about protein shape resemblance asks for a measure of the similarity of two protein surfaces. With this paper, we begin an investigation of this question. Our eventual aim is to get a meaningful measurement of the relative similarity of any pair of proteins. It seems useful however to first compare protein geometries to a single well understood benchmark. We could take some fixed protein as a benchmark, but the results we obtain would then be dependent on a rather arbitrary choice of a reference protein. To develop our method in a geometrically meaningful framework, we use the round sphere as a base shape to compare to a range of proteins. The sphere is the most symmetrical surface in 3-space, and the resemblance of a protein to a sphere reflects the symmetry, convexity, and globularity of the protein. With this in mind, we focus on the following question: How round is a protein? A suitable answer would assign a nonnegative number to each protein that indicates how far away it is from being round. This number should be stable under small perturbations, and not change significantly for different poses of a single flexible protein. We also choose it to be independent of scale.

Ideally, shape comparison techniques aim at defining directly a map between any two shapes that is as close to an isometry as possible. This is however a difficult problem, as the space of possible near-isometric maps is extremely large and not straightforward to characterize mathematically. Despite these difficulties, there have been many methods developed to find such mappings, including one for mapping bio-molecular surfaces onto the sphere (Rahi and Sharp, 2007). These methods rest on the definition of a distance measure that evaluates how close the map is to an isometry, on choices of sets of points on the two shapes, and on an algorithm for finding the mapping between these sets of points that minimizes this distance measure. The harmonic or Dirichlet energy (Eck et al., 1995; Alliez et al., 2002), the Procrustes distance and its continuous variant (Lipman et al., 2013a), the Gromov-Hausdorff distance and variants (Bronstein et al., 2006; Mémoli, 2007), and the conformal Wassterstein distance (Boyer et al., 2011; Lipman and Daubechies, 2011; Lipman et al., 2013b) are popular distance measures used in this context. The closest to-isometric mapping is then found by exhaustive evaluation of the chosen distance measure over all permutations of the landmark points on the two surfaces (Mémoli and Sapiro, 2005), by direct optimization, such as the generalized multi-dimensional scaling algorithm proposed by Bronstein and colleagues in (Bronstein et al., 2006), or through conformal parametrization of the surfaces (Gu and Yau, 2003; Gu et al., 2004).

In this paper we introduce a new method for measuring the similarity between a protein and the sphere that is based entirely on intrinsic geometry. It compares the two shapes by measuring the distortion of an optimal conformal mapping of the surface of one to the surface of the other. A preliminary report of this method was published in Koehl and Hass (2014). We assume that the surface of the protein is a surface of genus zero in ℝ^3^. This allows us to look for optimal diffeomorphisms (differentiable maps with differentiable inverses) between the surface and the sphere. The restriction to genus zero is appropriate for a wide variety of natural surface comparison problems, including facial recognition (Wang et al., 2005), alignment and comparison of brain cortical surfaces (see for example Gu et al., 2004; Hurdal and Stephenson, 2009), and geometric identification and comparison of bones (for example Boyer et al., 2011), in addition to protein surfaces (Rahi and Sharp, 2007). Compared to the other techniques for comparing genus zero surfaces mentioned above, the method we describe here has the advantage of being both computationally efficient and dependent only on the intrinsic surface geometry of the protein. Computational efficiency allows for comparisons with large collections of shapes, such as those found in the Protein Data Bank. Dependence on the intrinsic surface geometry makes our method well suited for modeling geometric similarities of flexible shapes, shapes that can bend over time to realize varying configurations in space. A substantial number of proteins demonstrate substantial flexibility, and thus our method seems well suited to their study.

As mentioned above, this paper is an extension of a previous study (Koehl and Hass, 2014). It differs mainly in that we have modified the elastic energy used to measure the difference between the optimal conformal mapping designed to map a surface onto another and an isometry, and we justify why. We also introduce a new quantitative measure of the similarity between a protein surface and the round sphere, and describe how this measure allows us to set the limits of the applications of conformal mapping to analyzing protein shapes. The paper is organized as follows. Section 2 provides the mathematical background for our algorithm: conformal geometry and measures of similarity between surfaces of genus zero. In Section 3, we provide the details of its implementation on discrete surfaces, as well as a description of the test cases used in the Results section. Section 4 presents and discusses the results obtained by our algorithm first on simple test cases to show the validity and power of the approach, then on a large dataset of proteins that are compared to the round sphere. We conclude the paper with a brief discussion on future developments.

## 2. Mathematical background

### 2.1. Basic idea: finding an optimal conformal mapping between two surfaces of genus zero

Let *F*_1_ and *F*_2_ be two surfaces of genus zero. Our goal is to define a map *f* : *F*_1_ → *F*_2_ that is as close as possible to an isometry, i.e., that minimizes the distortion of pairwise geodesic distances between points. When *F*_2_ = *S*^2^, i.e., the unit 2-sphere in ℝ^3^ and *F*_1_ and *F*_2_ are scaled to have the same area, then *f* gives a measure of the roundness of *F*_1_. We always in this paper scale two surfaces to have the same area, which we can take to be 4π, the area of the unit sphere. We then say that *F*_1_ is round if *f* is an isometry. For a surface that is not round, some metric distortion is found in any map to or from the sphere.

We now fix *F*_2_ = *S*^2^ to be isometric to the unit sphere. A deep result, the *Uniformization Theorem*, states that given any smooth genus zero surface *F*, there is always a conformal diffeomorphism from *F*_1_ to *S*^2^ (see Bers, 1972). Such conformal maps are not unique. Each conformal diffeomorphism *f* : *F*_1_ → *S*^2^ is part of a family of conformal diffeomorphisms. The space of conformal diffeomorphisms from *S*^2^ to itself forms the group PSL(2, ℂ), called the *Möbius* or *Linear-Fractional* transformations. Any conformal map *C* : *F*_1_ → *S*^2^ can be composed with a conformal Möbius transformation ϕ : *S*^2^ → *S*^2^ to give a new conformal map ϕ ◦ *C* : *F*_1_ → *S*^2^, and this construction gives all orientation-preserving conformal maps from *F*_1_ to *S*^2^.

**Figure 1:**
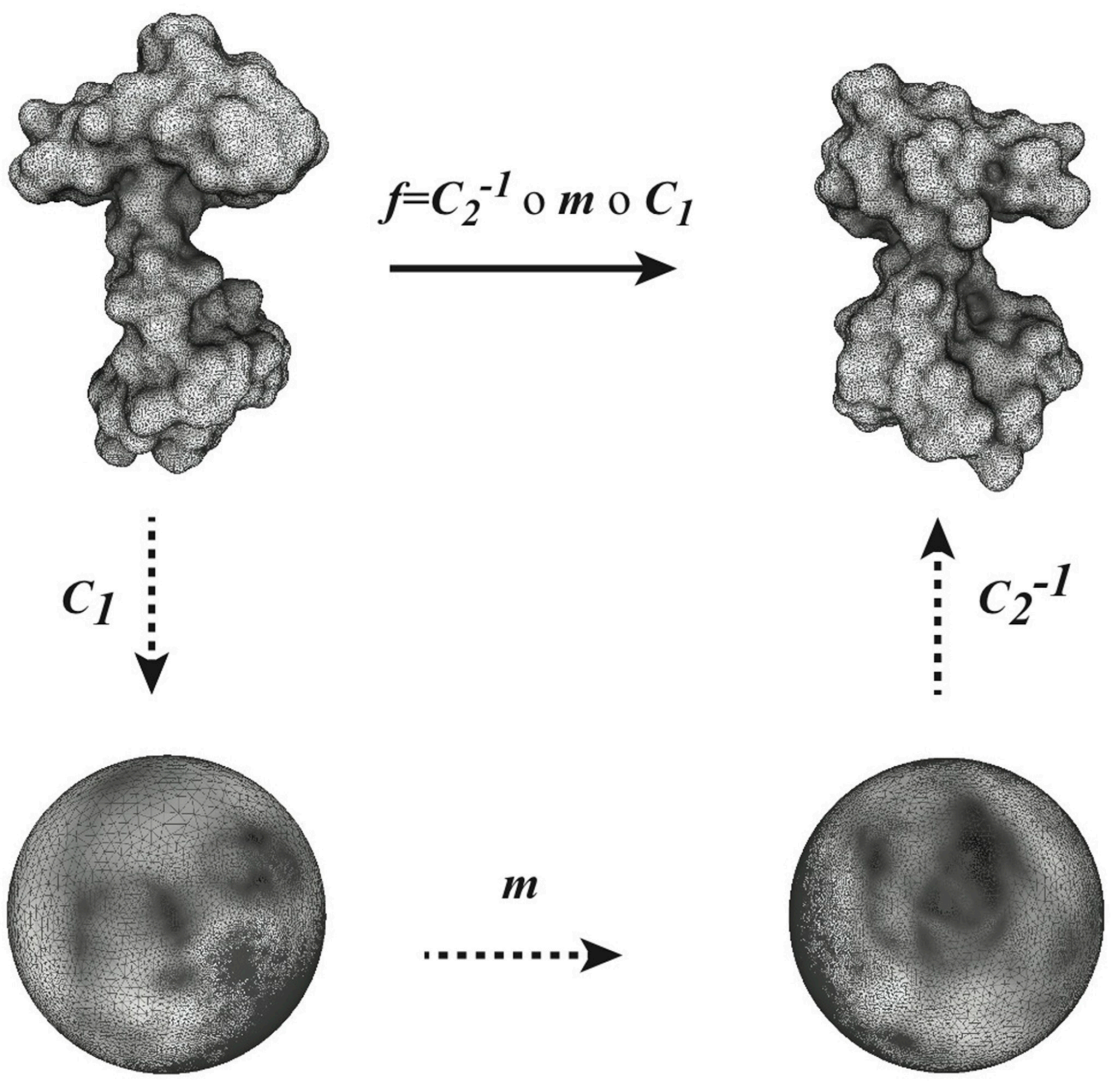
**Globally optimal conformal mapping**. The direct comparison of two surfaces *S*_1_ and *S*_2_ relies on the existence of a mapping *f* between these surfaces. In general a closed form for f is not known. When the two surfaces are of genus zero, it is however possible to construct *f* as a composition of three mappings *C*_1_, *m*, and *C*_2_, where *C*_1_ and *C*_2_ are conformal mappings from the surfaces *S*_1_ and *S*_2_ to the sphere and *m* is a bijective conformal mapping of the sphere to itself. The key to our approach is that the group of conformal self-mappings of the sphere is known: it is the group of Möbius transforms. As such, *m* is defined by six parameters that are optimized to yield minimal distortion (see text for details).

Given two surfaces *F*_1_ and *F*_2_ and a conformal mapping *f* between them, *f* can be understood as the composition of three conformal mapping functions, *C*_1_, *m* and *C*^−1^_2_ (see Figure 1). In this composition, *m* is a Möbius transformation that may arise through composition with transformations ϕ_1_ and ϕ_2_ as described above. We can choose *m* among the six-dimensional space of Möbius transformations to yield minimal distortion.

### 2.2. Distortion from an isometry

At a point *p* ∈ *F*_1_, a conformal map *f* : *F*_1_ → *F*_2_ stretches the metric of *F*_1_ uniformly in all directions by a positive factor λ(*p*). A conformal diffeomorphism then defines a real valued function λ : *F*_1_ → ℝ^+^ that measures this point-wise stretching. The function λ > 0 is called the *dilation* and is defined by the formula

(1) $$f^{*}(g_{2})=\lambda^{2}g_{1}$$

where *g*_1_, *g*_2_ are the metrics on *F*_1_, *F*_2_ respectively. Since λ > 0, it can be represented in the form λ = *e*^*u*^, where *u* : *F*_1_ → ℝ is a real-valued function.

We use the following energy function to measure the distortion of a conformal map *f* : *F*_1_ → *F*_2_ from an isometry. Recall that we have scaled all surfaces to have area equal to one.

**Definition**. The *symmetric elastic energy* of a conformal diffeomorphism *f* : *F*_1_ → *F*_2_ with dilation function λ = *e*^*u*^ is given by

(2) $$E_{S}(f)=E_{n}(f)+E_{n}(f^{-1})=\int_{F_{1}}u{\left(x\right)}^{2}dA+\int_{F_{2}}u{\left(y\right)}^{2}dA.$$

In (Koehl and Hass, 2014), we considered a different distortion energy function:

(3) $$E(f)=\int_{F_{1}}{\left(\lambda (x)-1\right)}^{2}dA.$$

Equations 2 and 3 differ at two levels. First, the distortion over a whole surface is computed using either the logarithm *u* of the dilation function λ, or λ directly. The latter varies between 0 and +∞, with values smaller than 1 corresponding to compression and values larger than 1 corresponding to expansion. As such, large compressions can contribute less to the total distortion than large dilations. In contrast, the function *u* = ln(λ) varies between −∞ and 0 for compression, and between 0 and +∞ for expansion, leading to a more balanced contribution for the two types of distortion. Second, *E*_*S*_(*f*) is symmetric and treats equally the distortions induced by *f* and those induced by *f*^−1^. In contrast, *E*(*f*) only accounts for the distortions induced by *f*. For these two reasons, we believe that *E*_*S*_(*f*) may be a better measure of distortion from an isometry.

The symmetric elastic energy defined in Equation 2 has the following properties (Hass and Koehl, in preparation):

1. For any pair of genus zero surfaces there is a smooth conformal homeomorphism between them that minimizes the symmetric elastic energy.
2. The symmetric elastic energy of a map is zero if and only if the map is an isometry. (Recall that we are assuming that all surface areas are equal to 4π.)

## 3. Materials and methods

### 3.1. A general algorithm for mapping two surfaces of genus zero

The algorithm described below is derived from our initial study of conformal mapping of genus zero surfaces described in Koehl and Hass (2014), which gives a comprehensive description. We focus here on the general concepts and on the differences with the original algorithm.

Let *F*_1_ and *F*_2_ be two surfaces of genus zero, represented by the meshes 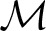
_1_ and 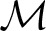
_2_, respectively. Both meshes are taken to be triangular, with 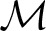
_*i*_ = (*V*_*i*_, *E*_*i*_, *T*_*i*_), *i* = 1, 2, where {*V*_*i*_, *E*_*i*_, *T*_*i*_} denote the vertices, edges and triangles, respectively. We note that these two meshes are completely independent of each other, and are likely to have different combinatorics.

As illustrated in Figure 1, we rely on the idea that a conformal mapping *f* between two surfaces *F*_1_ and *F*_2_ of genus zero can be written as the composition of two discrete conformal mappings *C*_1_ and *C*_2_ that parametrize *S*_1_ and *S*_2_ onto the sphere, and a Möbius transformation *m*. In optimizing the map produced from this composition, *C*_1_ and *C*_2_ are fixed, while *m* is variable and depends on six degrees of freedom, summarized in a parameter vector $\vec{h}$. The key to our approach is to choose the transformation *m* to minimize the sum of the distortions between the mesh 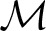
_1_ representing *F*_1_ and its image *W*_*m*_(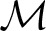
_1_) warped by *f* onto *F*_2_, and between the mesh 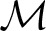
_2_ representing *F*_2_ and its image *W*^−1^_*m*_(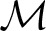
_2_) warped by *f*^−1^ onto *F*_1_. The total distortion is a discrete version of the symmetric elastic energy given by Equation 2 and is computed as a sum over all edges of the two surface meshes:

(4) $$\begin{array}{l} E_{S}(f)=\sum_{e_{ij}\;\in \;E_{1}}{\left(\ln\frac{{l^{\prime }}_{ij}}{l_{ij}}\right)}^{2}\frac{A_{ijk}+A_{ijm}}{3}\\ \;+\sum_{e_{kn}\;\in \;E_{2}}{\left(\ln\frac{{l^{\prime }}_{kn}}{l_{kn}}\right)}^{2}\frac{A_{knp}+A_{knq}}{3} \end{array}$$

Here *E*_1_, *E*_2_ denote the set of edges in the meshes on *F*_1_ and *F*_2_ respectively, *l*_*ij*_ denotes the length of the edge *e*_*ij*_ ∈ *E*_1_ that connects vertices *v*_*i*_, *v*_*j*_ and *l*′_*ij*_ the distance from *f*(*v*_*i*_) to *f*(*v*_*j*_). Similarly *l*_*kn*_ denotes the length of the edge *e*_*kn*_ ∈ *E*_2_ that connects vertices *v*_*k*_, *v*_*n*_ and *l*′_*ij*_ the distance from *f*^−1^(*v*_*k*_) to *f*^−1^((*v*_*n*_). The areas of the two triangles adjacent to the edge *e*_*ij*_ are given by *A*_*ijk*_ and *A*_*ijm*_. When *f* maps a pair of vertices *v*_*i*_, *v*_*j*_ of *F*_1_ to arbitrary points in *F*_2_, the distance between these points is computed by extending the metric on the edges of *F*_2_ to a flat Euclidean metric on each 2-simplex of the triangulation.

We have developed all the tools we need to search for a conformal map between two surfaces of genus zero that has minimal distortion, as defined by Equation 4.

- An algorithm for computing the discrete conformal mappings *C*_1_ and *C*_2_:
  While Riemann's Uniformization Theorem guarantees that any smooth genus zero surface *F* can be mapped conformally to the unit sphere, the theoretical underpinnings of the theory of discrete conformal maps are still being developed. Many methods have been developed to compute them in practice. We follow the approach proposed by Springborn and colleagues, which introduces a notion of discrete conformal equivalence (Springborn et al., 2008). In this method, the mesh 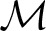 representing a genus zero surface *F* is first made topologically equivalent to a disk by removing a vertex *v*_0_ and its star. The transformed mesh is projected conformally on a plane through an optimization procedure (Springborn et al., 2008). The planar mesh is then warped onto the sphere by stereographic projection. Vertex *v*_0_ is reinstated on the North pole of the sphere and connected back to the mesh. Finally, we apply a Möbius normalization to ensure that the center of mass of all vertices is at the origin of the sphere. Full details on the implementation of this algorithm are provided in Koehl and Hass (2014).
- An algorithm for generating the warping of a discrete mesh onto a surface for a given Möbius transformation *m* : *S*^2^ → *S*^2^:
  This algorithm works as follows. A vertex *v*_*i*_ in 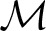
_1_ has image *v*′_*i*_ = *C*_1_(*v*_*i*_) in the spherical mesh *C*_1_(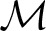
_1_). We locate the image *v*″_*i*_ = *m*(*v*′_*i*_) on the spherical mesh *C*_2_(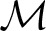
_2_), namely we identify the triangle *t* of *C*_2_(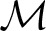
_2_) that contains *v*″_*i*_ and compute barycentric coordinates (α, β, γ) of *v*″_*i*_ in *t*. Finally, we compute the position of *v*‴_*i*_ = *f*(*v*_*i*_) on the surface *F*_2_ by propagating the barycentric coordinates (α, β, γ) onto the triangle *t*′ in 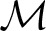
_2_ that corresponds to *t*. Full details on the implementation of this method are provided in Koehl and Hass (2014).

To simplify the notation, we write *E*_*S*_(*f*) = *E*_*S*_(*m*($\vec{h}$)) = *E*_*S*_($\vec{h}$) as the map *f* is determined by *m* which in turn is determined by the six parameters of $\vec{h}$. Simple calculations provide the analytical expressions for the symmetric elastic energy function *E*_*S*_($\vec{h}$) and its gradient with respect to $\vec{h}$. This allows us to apply a steepest descent algorithm to search for an optimum for the Möbius transformation *m*. Our general algorithm for comparing the two surfaces *F*_1_ and *F*_2_ represented with the discrete meshes 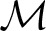
_1_ and 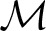
_2_ respectively, is then:

The scaling of the surface meshes in step (1) makes our comparison method insensitive to global changes of scale. While not necessary, this step is appropriate to measure scale invariant properties such as roundness. It is also appropriate when the global scale used to describe the vertex positions of the input surfaces is unknown. The damping parameter α_*n*_ in step (6) is obtained by solving the equation *E*_*S*_($\vec{h}$_*n*_ + α_*n*_∇*E*_*S*_($\vec{h}$_*n*_)) ≤ *E*_*S*_($\vec{h}$_*n*_) using a line search method. The value of TOL is set to a small constant related to machine error.

We have implemented the whole procedure outlined in Algorithm 1 into a Fortran program, RoundProtein. The results of a run of this program include a warping of the mesh 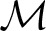
_1_ onto the surface *F*_2_, 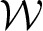
_2_(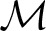
_1_) and its corresponding inverse, a warping of the mesh 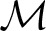
_2_ onto the surface *F*_1_, 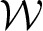
_1_(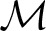
_2_), that minimizes distortion from an isometry among nearby conformal maps, as measured by the symmetric elastic energy. In addition, it gives a numeric measure of the geometric difference between 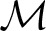
_1_ and 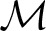
_2_ based on Equation 4. When the surfaces *F*_1_ and *F*_2_ are isometric, any energy minimizer is an isometry.

**Algorithm 1:** **Conformal mapping with minimal distortion between discrete surfaces of genus zero**.

**Initialization**. (1) Scale 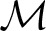 _1_, 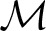 _2_ to have total area one.
(2) Find *C*_1_ and *C*_2_ that conformally map 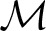 _1_ and 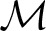 _2_ onto the sphere, using the method described above.
(3) Initialise Möbius transformation *m*_0_ = *m*($\vec{h}$_0_).
**for** *n* = 0, … until convergence
(4) Generate *f*( 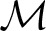 _1_) and *f*^−1^( 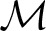 _2_) using the warping method described above, where *m* = *m*($\vec{h}$_*n*_).
(5) Compute *E*_*S*_($\vec{h}$_*n*_) and its gradient ∇*E*_*S*_($\vec{h}$_*n*_) with respect to $\vec{h}$_*n*_.
(6) Update $\vec{h}$_*n*+1_ = $\vec{h}$_*n*_ − α_*n*_∇*E*_*S*_($\vec{h}$_*n*_).
(7) Check for convergence: if *E*_*S*_($\vec{h}$_*n* + 1_) <TOL, stop.
**end for**

When *F*_2_ is set to be the round sphere, *d*(*F*_1_, *S*^2^) is a measure of the roundness of the surface *F*_1_.

### 3.2. Triangular meshes for regular shapes

To compare surfaces of genus zero to the round 2-sphere *S*^2^, we need a triangular mesh 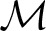
(*S*^2^). We generate 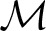
(*S*^2^) by positioning *N* points uniformly on the sphere and forming a triangulation from these *N* points.

Distributing points uniformly on the 2-sphere is one of eighteen unsolved mathematics problems proposed by the mathematician (Smale, 1998). We adopt the Thompson formulation of this problem and define it as the problem of determining the minimum electrostatic potential energy configuration of *N* electrons on the surface of a unit sphere, that repel each other with a force given by Coulomb's law, (Thomson, 1904). The total electrostatic potential energy of a N-electron configuration is expressed as the sum of all its pair-wise interactions,

(5) $$U(N)=\frac{1}{4\pi \epsilon_{0}}\sum_{i<j}\frac{1}{\left\|r_{i}-r_{j}\right\|}$$

where ϵ_0_ is the vacuum permittivity and *r*_*i*_ is the coordinate vector of electron *i*. A minimum value of *U*(N) over the configurations of *N* distinct points is found by numerical minimization. We used for this the Matlab package “Uniform sampling of the sphere” available from Semeshko (2012). Once a minimum configuration is obtained, a triangular mesh is generated using QHull (Barber et al., 1996). We note that the optimization of *U*(N) is computationally intensive. To generate a mesh that is dense enough on the sphere, we have used the method described here for *N* = 1000 and subdivided the corresponding mesh recursively using triangular quadrisection (in this process, a triangle is subdivided into 4 triangles by adding the three edges that join the midpoint of its three sides).

In parallel, we have generated dense triangular meshes of the surfaces of the Platonic solids using a similar procedure. Starting from the vertices of a platonic solid, we generate a triangular mesh using QHull. This mesh is then subdivided recursively using triangular quadrisection.

Table 1 summarizes the characteristics of the triangular meshes generated for the sphere and the five Platonic solids.

**Table 1:** **Characteristics of the discrete meshes of regular shapes**.

**Shape**	**Vertices**	**Faces**	**Vertices in fine mesh**	**Faces in fine mesh**
Tetrahedron	4	4	8194	16,384
Cube	8	6	6146	12,288
Octahedron	6	8	16,386	32,768
Dodecahedron	20	12	18,434	36,864
Icosahedron	12	20	10,242	20,480
Sphere			15,970	31,936

### 3.3. Data set of protein structures

The set of structures considered in this study is extracted from the database of 2930 sequence-diverse CATH (Orengo et al., 1997) v2.4 domains used in a previous study (Kolodny et al., 2005). As we focus on three-dimensional structures, we consider the first three levels of CATH, Class, Architecture and Topology, to give a CAT classification. We refer to a set of structures with the same CAT classification as a *fold*. We selected five of the most populated folds in the database of 2930 structures as the test set for all computational experiments run in the studies presented in this paper, including at least one fold from each CATH class: CATH fold 1.10.10, a fully α fold (arc repressor, 55 representatives), CATH fold 2.60.40, a fully β fold (immunoglobulin-like, 156 representatives), and three mixed α−β folds: 3.20.20, (TIM-like, 52 representatives), 3.30.70, (two layer sandwich, 85 representatives) and 3.40.50 (Rossmann fold, 185 representatives). These five folds include a total of 533 proteins.

We represent the surface of each protein by its skin surface (Edelsbrunner, 1999), given as a triangulated mesh that surround the atoms of the protein. We use the standard model in chemistry of representing a protein structure as a union of balls, with each ball corresponding to an atom. The skin surface of a protein is then computed from the boundary of the union of these balls, where the center of a ball is given by the coordinates of the corresponding atom, and its radius is set to 2^1/6^σ + *R*_*H*2*O*_, where σ is the vdW parameter for the atom in the AMBER94 force field (Cornell et al., 1995) and *R*_*H*2*O*_ is the radius of the solvent probe, set to 1.4 Å.

We generated high quality meshes for the skin surfaces of all 533 proteins using the program smesh, described in detail in Cheng and Shi (2004, 2009). Briefly, the algorithm implemented in smesh uses a Delaunay-based method to generate quality mesh for the skin surface incrementally. In particular, points are sampled one by one on the skin surface using a front advancing method. The Delaunay triangulation of the sample points is maintained using an incremental flipping algorithm developed by Lawson (1972). A subset of the Delaunay triangulation is extracted that defines candidate surface triangles. These candidate surface triangles form a partial mesh and guides the subsequent point samplings. The procedure is applied iteratively until an ϵ-sampling of the whole surface is obtained. The corresponding surface triangles define the skin surface mesh. The corresponding triangular meshes have similar sizes for all proteins, with approximately 25,000 vertices and 50,000 triangles on average We checked that all the meshes have genus zero.

## 4. Results and discussion

### 4.1. How round are the platonic solids?

We first consider the surfaces formed by the boundaries of the five Platonic solids: the tetrahedron (4 faces), the hexahedron, or cube (6 faces), the octahedron (8 faces), the dodecahedron (12 faces), and the icosahedron (20 faces). These highly symmetric surfaces serve as a collection of coarse to fine discrete representations of the sphere, with known measures of the quality of the approximation. As such, they provide natural test cases for the effectiveness of our approach to measure surface roundness.

**Figure 2:**
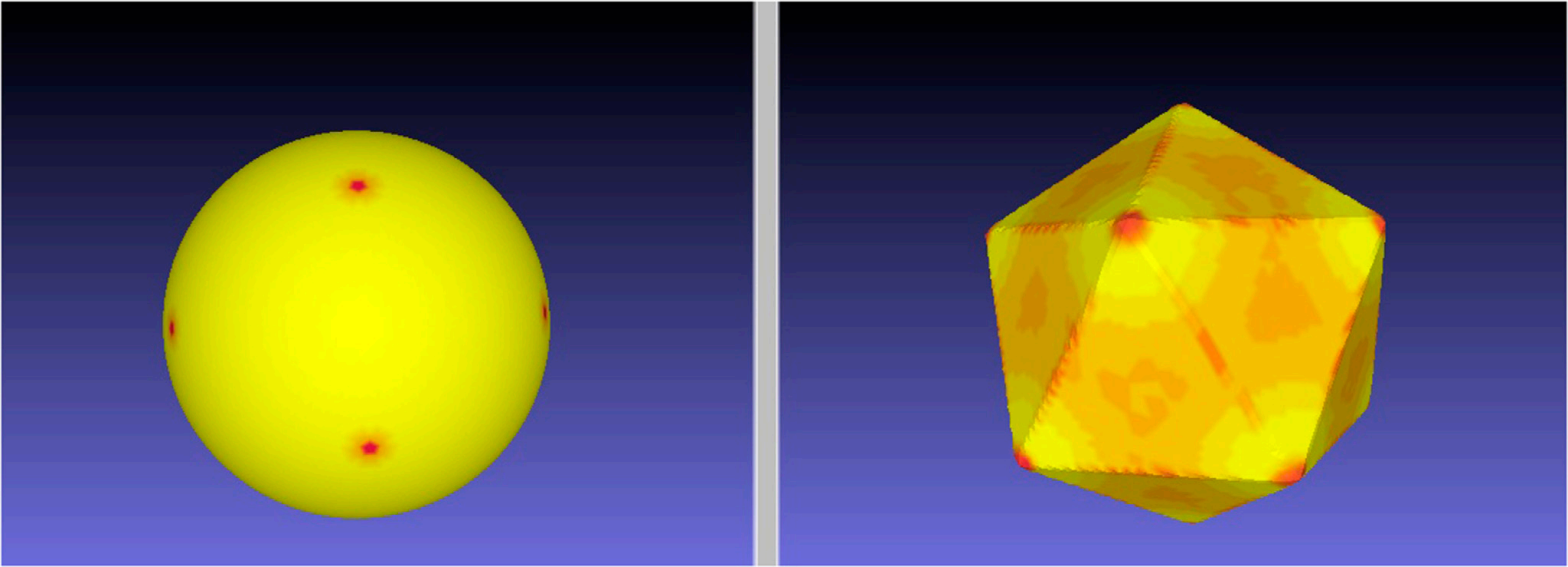
**An *E*_*S*_ minimizing map between the sphere and the icosahedron**. We computed the minimal distortion conformal map between the discrete mesh 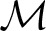
_1_ representing the icosahedron and the discrete mesh 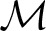
_2_ representing the sphere, where distortion is defined by the symmetric elastic energy given by Equation 4. The left panel shows the warping of the mesh 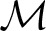
_1_ onto the surface of the sphere, while the right panel shows the warping of the mesh 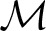
_2_ onto the surface of the icosahedron. Red on the target indicates large dilation in the source.

Figure 2 illustrates the quality of the optimal mapping obtained with RoundProtein between the sphere and the icosahedron, both represented with fine discrete triangular meshes whose characteristics are given in Table 1. The resulting warping of the icosahedron mesh onto the surface of the sphere shows 12 dense spots, corresponding to the 12 vertices of the icosahedron (left panel). In contrast, the warping of the discrete mesh representing the sphere onto the surface of the icosahedron shows smaller distortion. It represents the icosahedron surface well, with relatively large dilation at the vertices (red spots on the right panel of Figure 2). These dilations are expected as the mesh of the sphere needs to adapt to the angle defect at these vertices. Similar results were observed for the four other Platonic solids (results not shown).

Two common measures of the roundness of a surface *F* ⊂ ℝ^3^ can also be computed analytically for the Platonic solids:

- The *sphericity* of a surface measures how efficiently the surface encloses volume. It is given as the ratio of the surface area of a sphere (with the same volume enclosed by the surface *F*) to the surface area of *F*:
  $$Sph=\pi^{1/3}{\left(6V\right)}^{2/3}/A,$$
  where *V* is the volume enclosed and *A* is the surface area. The sphericity is at most one, and equals one only for the round sphere.
- The ratio *R*_*IC*_ of the radii of inscribed and circumscribed spheres. This is often used as a measure of roundness for convex surfaces, but is less useful for general shapes.

We will compare these roundness measures with *E*_*S*_. Note however that these measures are *extrinsic*, depending on the particular embedding of a surface into ℝ^3^. They will not be preserved under flexing and bending, unlike *E*_*S*_.

In addition, we can measure local deformations between a Platonic solid and the sphere by computing the solid angle Ω at each vertex. The solid angle Ω is given by

(6) Ω=qθ−(q−2)π,

where

(7) $$\sin\frac{\theta }{2}=\frac{\cos(\pi /q)}{\sin(\pi /p)}.$$

θ is the interior angle between any two face planes of the solid, *p* is the number of edges of each face, and *q* is the number of faces meeting at each vertex.

In Table 2 we report the values of these measures of roundness for all five Platonic solids as well as the minimal symmetric elastic energies obtained when computing the conformal mapping between the solids and the sphere using RoundProtein. As expected, the sphericity, *R*_*IC*_, and the solid angles Ω increase as the number of faces of the solid increases, i.e., as the solid becomes a better approximation of the sphere. In parallel, *E*_*S*_ decreases, i.e., the differences between the conformal mapping constructed between the solid and the sphere and the isometry become smaller as the number of faces increases. The decrease in *E*_*S*_ is highly correlated with the increases in sphericity, *R*_*IC*_, and solid angles, with Pearson's correlation coefficients of −0.92, −0.92, and −0.84, respectively.

**Table 2:** **Roundness of the Platonic solids**.

**Surface**	**# of faces**	**Sphericity, *Sph***	***R*_*IC*_**	**Solid angle, Ω**	***E*_*S*_**
Tetrahedron	4	0.671	$\frac{1}{3}\approx 0.333$	0.551	0.96
Cube	6	0.806	$\frac{1}{\sqrt{3}}\approx 0.577$	1.571	0.12
Octahedron	8	0.846	$\frac{1}{\sqrt{3}}\approx 0.577$	1.360	0.24
Dodecahedron	12	0.910	0.795	2.962	0.02
Icosahedron	20	0.939	0.795	2.635	0.04
Sphere	Not defined	1.000	1.000	Not defined	0.00

**Figure 3:**
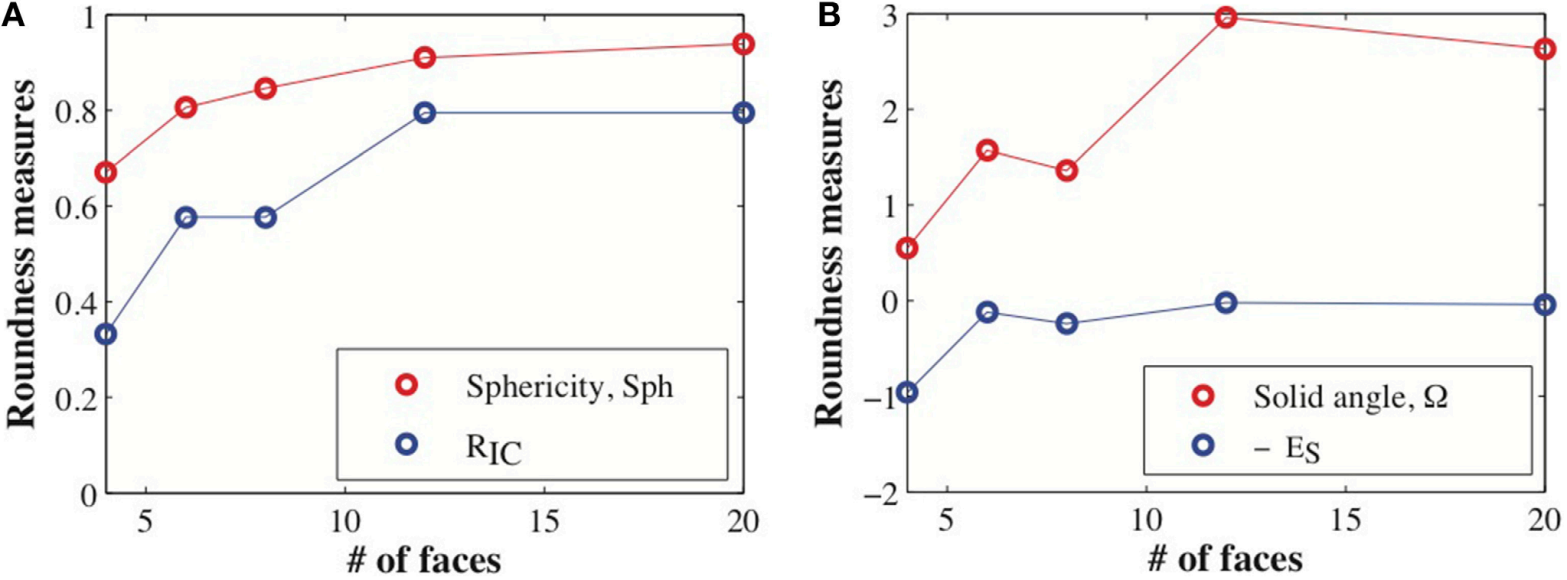
**Global and local measures of roundness for the Platonic solids**. We computed the sphericity, *S*ph, ratio of the radii of the inscribed and circumscribed sphere, *R*_*IC*_, solid angle Ω at the vertices, and symmetric elastic energy *E*_*S*_ of minimal distortion conformal map between the Platonic solids and the sphere. **(A)** Both *S*ph and *R*_*IC*_ vary monotonically with the number of faces of the solid, slowly converging to the expected value of 1 for a sphere. **(B)** F both Ω and *E*_*S*_ (shown as −*E*_*S*_ for clarity), we observe two inversions (i.e., non monotonic behavior) when compared to the number of faces: the cube and the octahedron, and the dodecahedron and icosahedron.

We note that the order of the different measures of roundness does not precisely coincide. *S*ph and *R*_*IC*_ increase monotonically as the number of faces increases. These two measures capture the global shape of the solid. In contrast, the solid angle Ω shows a non-monotonic behavior, illustrated in Figure 3. Ω is a measure of local differences with the sphere, as it measures how the local shape around a vertex of the solid differs from a round sphere. While the octahedron has more faces than the cube, its vertices have a smaller solid angle, i.e., they have less local resemblance to the sphere. The same difference in ordering is observed between the dodecahedron and the icosahedron. Interestingly, the symmetric elastic energy *E*_*S*_ captures these local differences between the shapes, while still decreasing as a shape gets closer globally to the sphere. As such, *E*_*S*_ is able to capture both local and non-local differences between a surface and a sphere.

### 4.2. How round is a protein?

Proteins come in a wide variety of sizes and shapes. Fibrous proteins, such as collagens that are important for structuring cellular tissues, have elongated shapes while globular spheroproteins that are responsible for catalyzing chemical reactions within cells adopt a compact structure. Understanding the relationship between a protein sequence, its shape, and its function is one of the fundamental problem in biology. Here we address a very specialized question within this problem, namely the characterization of the globularity of a protein, or a quantification of its roundness. A protein structure can be depicted in many different ways, each emphasizing different features of the protein. We focus on the geometry of a 2-dimensional surface that encloses the protein, as defined by the skin surface (Edelsbrunner, 1999).

**Figure 4:**
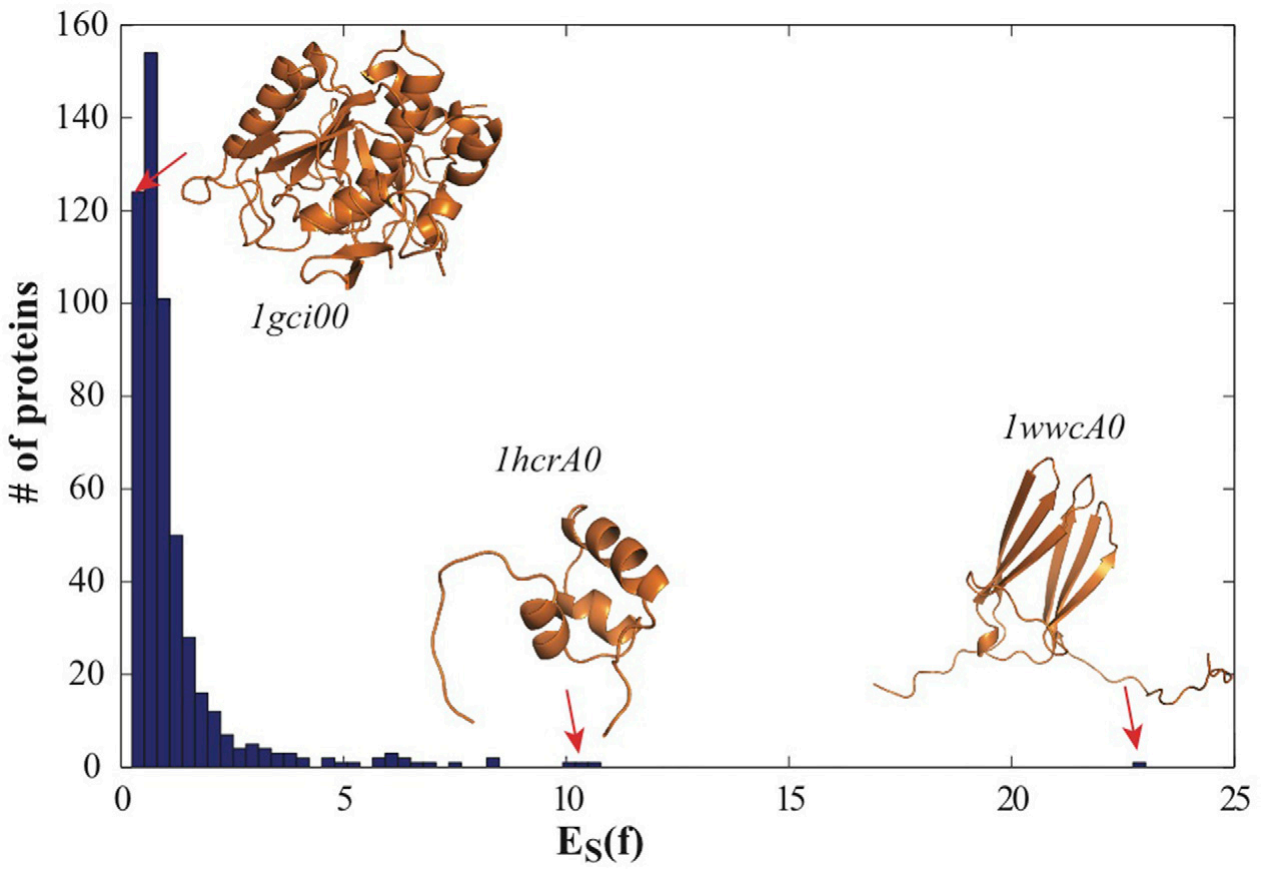
**The distribution of the optimized symmetric elastic energies *E*_*S*_ for the 533 proteins in CATH533**. Proteins 1gci00, 1hcrA0 and 1wwcA0 are highlighted as they correspond to the proteins with the lowest (0.24), second to highest (10.2) and highest (23.0) symmetric elastic energies, respectively.

We use CATH533 as our data set of proteins to assess our approach to measuring the roundness of a surface. CATH533 is a database of 533 protein structures that covers the three main classes of CATH: one fully α fold, one fully β fold, and three α − β folds (the TIM fold, an α/β plait, and the Rossmann fold) (see Materials and Methods section above for details). We generated a mesh for each protein in CATH533 using the program smesh (Cheng and Shi, 2004, 2009) and computed the optimal conformal mapping between this corresponding mesh and the discrete mesh representing the 2-sphere using RoundProtein. In Figure 4, we show the distribution of corresponding optimized symmetric elastic energies *E*_*S*_.

All proteins included in CATH533 are enzymes and therefore they are expected to be globular. Indeed, we observe that computing the optimal mappings *f* between these proteins and the sphere leads to mappings that are close to isometries, as measured by *E*_*S*_(*f*), the symmetric elastic energy of the optimal mapping given in Equation 4. Of the 533 proteins, 352 have an optimized *E*_*S*_(*f*) below 1, and 106 of those have an optimized *E*_*S*_(*f*) below 0.5. The “best" mapping, i.e. the one closest to an isometry, is observed for the protein with CATH code 1gci00. The latter corresponds to PDB code 1gci which contains the ultra-high resolution (0.78 Å) of *B. Lenti* subtilisin, a serine protease that is known to form a very compact beta barrel at its core (Kuhn et al., 1998). The corresponding optimized symmetric elastic energy of 0.24 would make this serine protease similar to an octahedron when compared to the sphere (see Table 2). The “worst” mapping, i.e., the least similar to an isometry, with an optimized symmetric elastic energy of 23.0, is observed for the protein with CATH code 1wwcA0. This is chain A from the PDB file 1wwc that contains the crystal structures of the neurotrophin-binding domains TrkA, TrkB, and TrkC, with chain A corresponding to TrkA. The TrkA domain is known to fold into an immunoglobulin-like structure, with a core of β-sheet and two long loops at the N and C termini (Ultsch et al., 1999). It is the presence of these two long loops that makes the structure deviate significantly from the sphere (see insert in Figure 4). Interestingly, the next to worst comparison of a protein surface with the 2-sphere is observed for the protein with CATH code 1hcrA0. This is chain A from the PDB file 1hcr, corresponding to the complex of a prokaryotic Hin recombinase bound to DNA. The recombinase adopts a 3 helix-bundle conformation, with two long flanking extended polypeptide regions that contact bases in the minor groove of the DNA (Feng et al., 1994). As we only consider the structure of the recombinase, these two regions stand aside from the core helix bundle, leading to a less compact structure (see insert in Figure 4).

**Figure 5:**
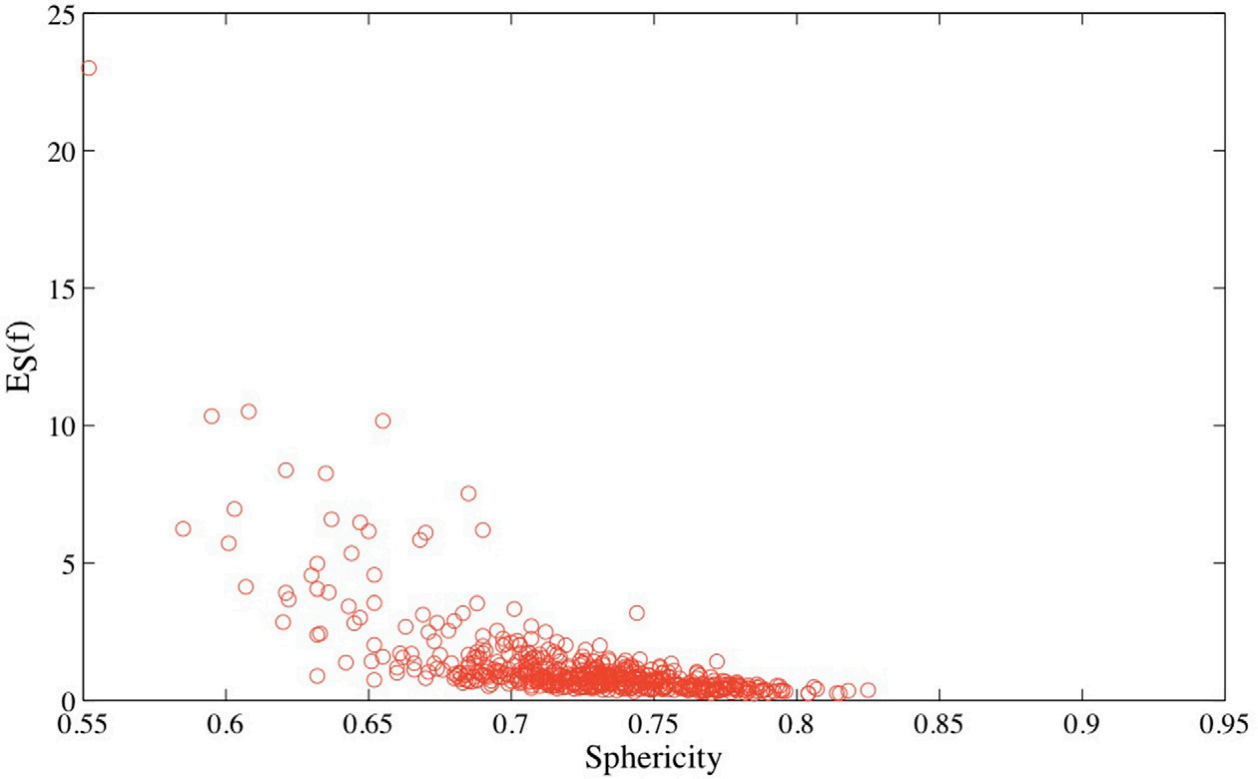
**The optimized symmetric energy of the conformal mapping between the surface of a protein and the round sphere, *E*_*S*_, vs. the sphericity of the protein surface, for all 533 proteins in CATH533**.

In Figure 5, we compare the optimized symmetric energy *E*_*S*_(*f*) of the mapping *f* between a protein surface and the sphere with the sphericity of the protein surface, computed using Equation 6, for all proteins in CATH533. Just as for the Platonic solids, *E*_*S*_(*f*) and the sphericity *S*ph are correlated: as the sphericity increases, the mapping between the protein surface and the sphere improves, and *E*_*S*_(*f*) decreases. Interestingly, the correlation coefficient between *E*_*S*_(*f*) and *S*ph for protein surfaces, –0.64, is significantly lower than the corresponding correlation coefficient for the Platonic solids, −0.92. We assign this difference to the fact that the latter are convex while the geometry of even globular proteins is more diverse, with more significant local differences to a round surface that are not captured by sphericity.

**Figure 6:**
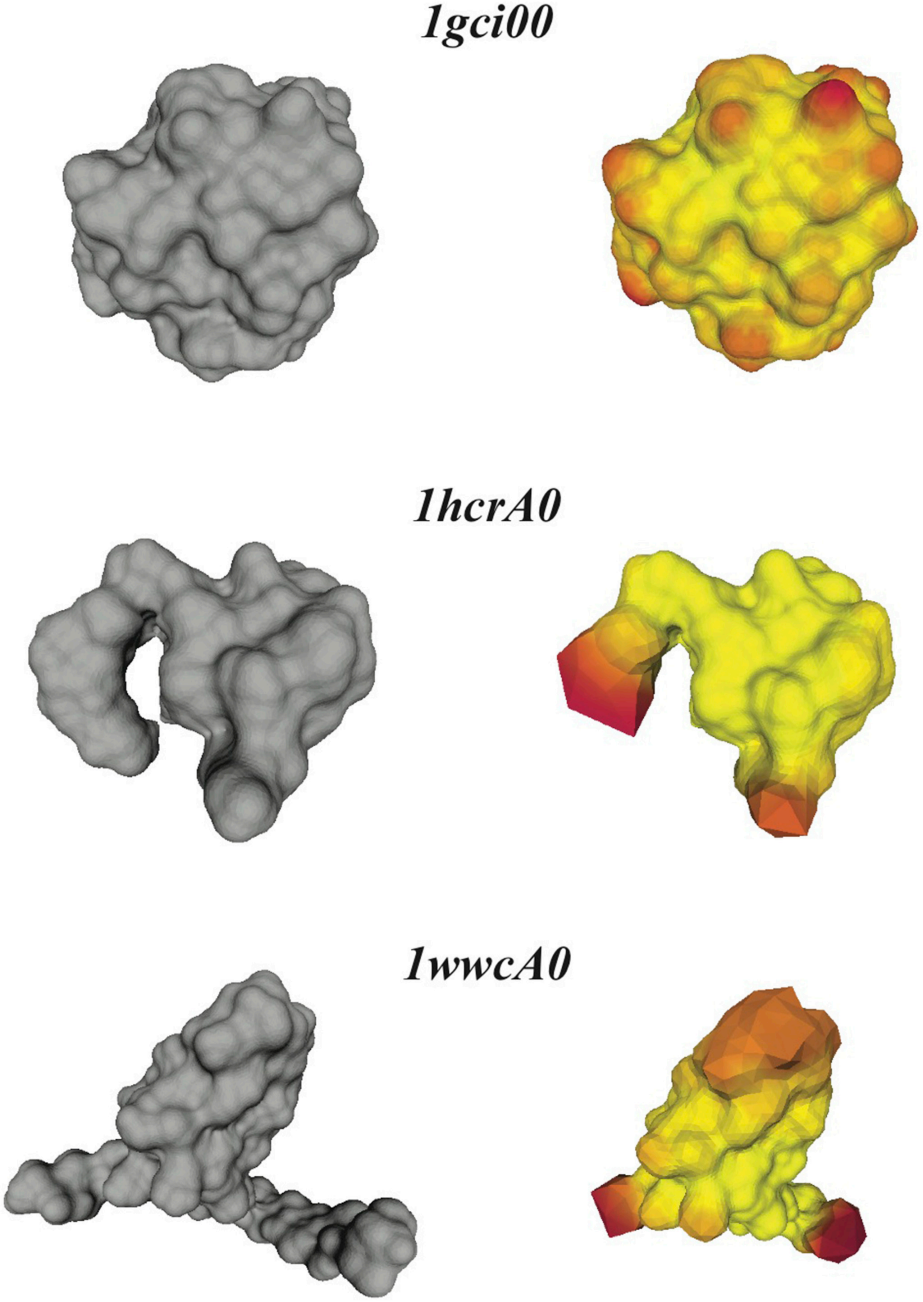
**Distortions in the conformal maps between protein surfaces and the sphere**. For the three proteins 1gci00, 1hcrA0, and 1wwa0 (see text for details), we compare their discrete skin surfaces (left panels), with the optimized surfaces generated from the conformal warping of the mesh representing the sphere onto the skin surfaces (right panels). Red on the warped surface indicates large distortions of the source mesh.

Figure 4 illustrates that the optimal conformal mapping between a protein surface that has long protruding regions and the sphere deviates significantly from an isometry. To help understand why this is the case, we compare in Figure 6 the surfaces of the three representative proteins identified in Figure 4 with the surfaces generated from the corresponding warping *f*^−1^(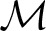
(*S*^2^)) of the mesh represented the sphere onto the surfaces of the three proteins, where the warping is generated with RoundProtein.

If the conformal mapping between a protein surface and the sphere is close to an isometry, it is expected that *f*^−1^(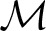
(*S*^2^)) closely follows the surface of the protein. This is indeed observed for the very compact protein 1gci00. The main distortions observed in the warped mesh occur at bumps in the surface (which correspond to the spherical representations of the atoms at the surface of the protein). In the case of the less compact proteins 1hcrA0 and 1wwcA0 however, the warped surfaces generated from *f*^−1^(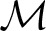
(*S*^2^)) deviate significantly from the actual surfaces of the proteins. Most of the distortions occur at the protruding regions that are not present in the images of the spheres on the protein surfaces. The discrete conformal mappings of these protruding regions to the sphere introduce very large negative conformal factors on their vertices, which in turn lead to infinitesimally small edge lengths in the projected meshes and consequently large numerical errors. We have observed similar behaviors when computing conformal mappings between generic genus zero surfaces (Koehl and Hass, 2014). This problem is not specific to our method, as it appears in many conformal mapping procedures. In some cases approximating by a conformal map appears to be too restrictive. One solution is to introduce cone singularities in the regions with the worst distortions (see for example Springborn et al., 2008).

**Figure 7:**
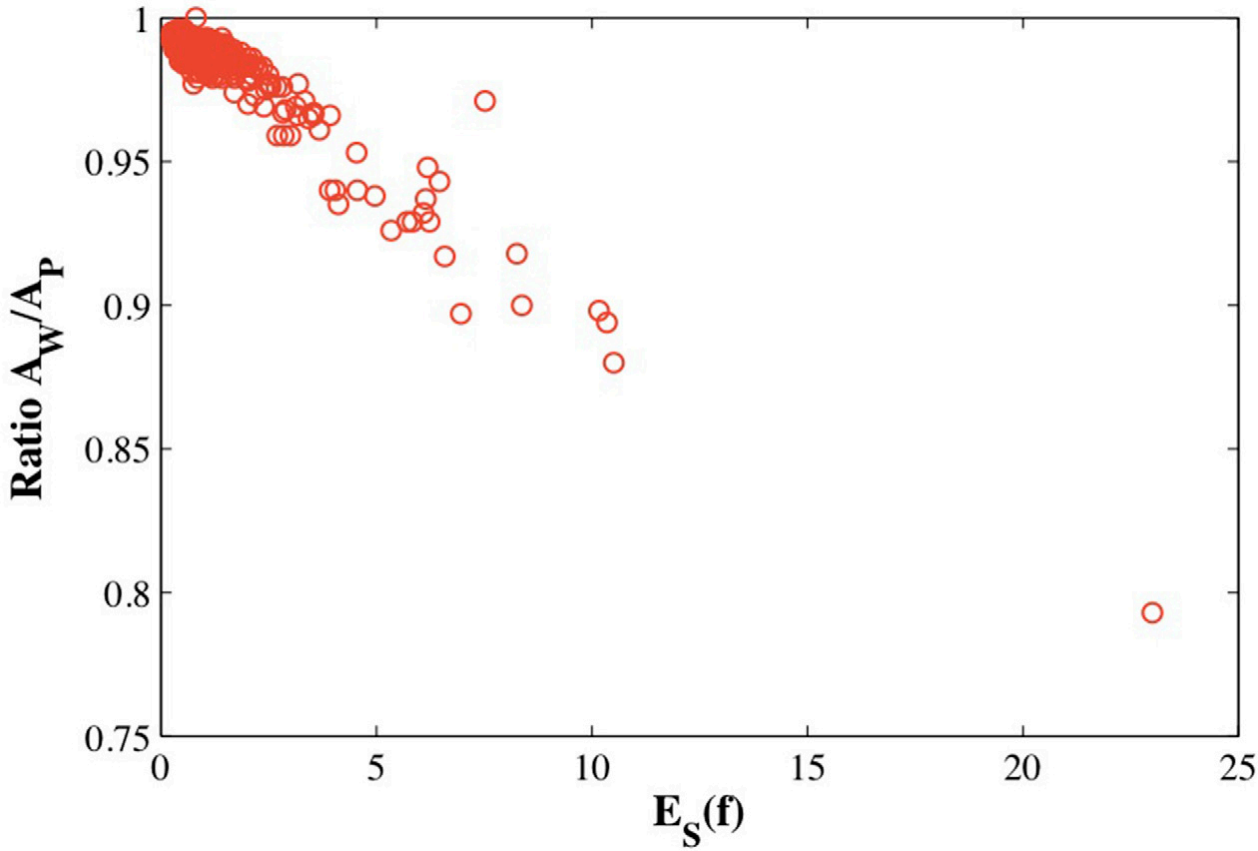
**The estimated distortion of the image *f*^−1^(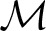
(*S*^2^) of the mesh of the sphere onto the surface of a protein, measured as the ratio of the surface area of this image and the surface area of the mesh representing the protein is plotted against the optimized symmetric energy of the conformal mapping *f*, *E*_*S*_(*f*)**.

Figure 6 illustrates that the distortions introduced by the restrictive condition that the mapping between the protein surface and the sphere be conformal lead to an image *f*^−1^(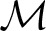
(*S*^2^)) of the mesh of the sphere onto the surface of the protein that does not capture well the geometry of this surface. One approach to measuring these distortions is to compute the ratio of the surface area *A*_*W*_ of *f*^−1^(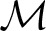
(*S*^2^)) to the surface area *A*_*P*_ of the source mesh representing this protein. We plot this ratio against the symmetric elastic energy of the refined mapping *f*, *E*_*S*_(*f*), in Figure 7 for all 533 proteins in CATH533. If the mapping *f* is close to an isometry, there should be minimal distortion and *f*^−1^(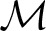
(*S*^2^)) should be a good representation of the surface of the protein (as illustrated in Figure 6 for 1gci00). The ratio *A*_*W*_/*A*_*P*_ should then be close to 1. This is indeed observed for the majority of the proteins in CATH533. We find that *A*_*W*_/*A*_*P*_ is greater than 0.99 for 226 proteins, greater than 0.98 for 471 proteins, and greater than 0.95 for 512 proteins. This ratio decreases significantly as *f* deviates more and more from an isometry, with a minimal value of 0.79 for protein 1wwcA0. Interestingly, *A*_*W*_/*A*_*P*_ and *E*_*S*_(*f*) are strongly correlated with a Pearson's coefficient of correlation of 0.95. This indicates that *E*_*S*_(*f*) has value as a tool to test whether a conformal map is accurately representing a given surface.

## 5. Summary and conclusions

We have developed a new method for quantifying the compactness of a protein structure. In this new approach we compute the conformal map *f* between the surface of the protein (required to be of genus zero) and the 2-sphere that has minimal distortion, where distortion is defined as a symmetric elastic energy *E*_*S*_(*f*) that measures the distance between *f* and an isometry. It leads to flexible registration of the two surfaces and accurate measurements of their geometric dissimilarities. Its implementation within the program RoundProteins is based on fast and robust numerical methods, making surface comparisons feasible for large data sets of proteins. We have illustrated its use for quantifying the roundness of the Platonic solids and of 533 diverse protein structures. We have demonstrated that the elastic energy *E*_*S*_(*f*) captures both global and local differences between two surfaces. We have shown that our method identifies and measures the presence of protruding regions in protein structures that make them deviate from a compact shape.

This paper is a first step toward achieving automatic registration of protein structures based on their surfaces. The method described here is an extension of the approach described in Koehl and Hass (2014) and suffers from similar limitations. We note that it only applies to surfaces of genus zero and that it works best for surfaces that have uniform geometry, without long protrusions (Koehl and Hass, 2014). In this paper, we have shown that this limitation can be used to generate valuable information. The difficulty that RoundProtein encounters in finding a conformal mapping *f* between a highly non-spherical protein surface and the 2-sphere translates into a high value for the symmetric elastic energy *E*_*S*_ of *f*. Such a high value measures the extent of the deviation of the protein from being approximately round. It also indicates the limits of the application of conformal mapping to parametrize protein shapes, as high values for *E*_*S*_ correspond to significant deviations between the representations of a surface given by its source mesh and the representation given by the parametrization formed by the target mesh (see Figure 6). For the limitation to genus zero surfaces, we note that the concept of discrete conformal structures can be extended to surfaces with arbitrary topology, either through the introduction of cone singularities (Springborn et al., 2008), or through the definition of a discrete conformal equivalence between a Euclidean triangulation on the surface and a flat or hyperbolic triangulation (Bobenko et al., 2010; Tsui et al., 2013). Finding closest-to-isometric mappings for surfaces with genus greater than zero remains a topic for future studies.

Finally, we note that while the symmetric elastic energy of a conformal mapping between two surfaces *F*_1_ and *F*_2_ defined in Equation 2 is useful for measuring the differences between these two surfaces, it is not clear that it establishes a distance on the space of genus zero shapes. A number of important applications would benefit from an actual metric on the space of genus zero surfaces.

## Author contributions

The two authors contributed equally to the work, as well as to the draft and following revisions of the manuscript.

### Conflict of interest statement

The authors declare that the research was conducted in the absence of any commercial or financial relationships that could be construed as a potential conflict of interest.
